# Effective Connectivity within the Default Mode Network: Dynamic Causal Modeling of Resting-State fMRI Data

**DOI:** 10.3389/fnhum.2016.00014

**Published:** 2016-02-01

**Authors:** Maksim G. Sharaev, Viktoria V. Zavyalova, Vadim L. Ushakov, Sergey I. Kartashov, Boris M. Velichkovsky

**Affiliations:** ^1^National Research Centre “Kurchatov Institute”Moscow, Russia; ^2^Faculty of Physics, M.V. Lomonosov Moscow State UniversityMoscow, Russia; ^3^Institute for Higher Nervous Activity and Neurophysiology, Russian Academy of SciencesMoscow, Russia; ^4^National Research University Higher School of EconomicsMoscow, Russia; ^5^Department of Cybernetics, National Research Nuclear University “MEPhI”Moscow, Russia; ^6^NBICS-Faculty, Moscow Institute of Physics and TechnologyMoscow, Russia

**Keywords:** effective connectivity, default mode network (DMN), resting-state fMRI, dynamic causal modeling (DCM), resting-state networks

## Abstract

The Default Mode Network (DMN) is a brain system that mediates internal modes of cognitive activity, showing higher neural activation when one is at rest. Nowadays, there is a lot of interest in assessing functional interactions between its key regions, but in the majority of studies only association of Blood-oxygen-level dependent (BOLD) activation patterns is measured, so it is impossible to identify causal influences. There are some studies of causal interactions (i.e., effective connectivity), however often with inconsistent results. The aim of the current work is to find a stable pattern of connectivity between four DMN key regions: the medial prefrontal cortex (mPFC), the posterior cingulate cortex (PCC), left and right intraparietal cortex (LIPC and RIPC). For this purpose functional magnetic resonance imaging (fMRI) data from 30 healthy subjects (1000 time points from each one) was acquired and spectral dynamic causal modeling (DCM) on a resting-state fMRI data was performed. The endogenous brain fluctuations were explicitly modeled by Discrete Cosine Set at the low frequency band of 0.0078–0.1 Hz. The best model at the group level is the one where connections from both bilateral IPC to mPFC and PCC are significant and symmetrical in strength (*p* < 0.05). Connections between mPFC and PCC are bidirectional, significant in the group and weaker than connections originating from bilateral IPC. In general, all connections from LIPC/RIPC to other DMN regions are much stronger. One can assume that these regions have a driving role within the DMN. Our results replicate some data from earlier works on effective connectivity within the DMN as well as provide new insights on internal DMN relationships and brain’s functioning at resting state.

## Introduction

One of the exiting discoveries in modern cognitive neuroscience, which was anticipated by the founding fathers of electrophysiology, is that our brain is never at rest. During wakeful periods when we are trying not to do anything, global metabolism does not decrease in the brain, and some of its distinct areas are still active. These areas constitute networks of the resting state conditions or RSNs. Moreover, during overall deliberate inactivity and inattention to the external world, small but consistent increases in activity occur in a specific set of regions called the “Default Mode Network” or DMN. It is a core part of a number of RSNs which were intensively investigated in the past couples of decades. Motor, visual, auditory networks and networks connected with language, working memory and attention were identified. It is believed that DMN, which was one of the first discovered, is closely related to the function of consciousness.

The default-mode activity is specific not only to the human species and non-human primates, but it is also present in rodents (Mantini et al., 2011; Mantini and Vanduffel, 2013). Therefore, DMN could be considered as one of the evolutionarily old conservative systems (Mantini et al., 2011; Mantini and Vanduffel, 2013). The DMN includes the posterior cingulate cortex/precuneus (PCC), medial prefrontal cortex (mPFC), bilateral inferior parietal lobule (IPL), and other regions including the inferior temporal gyrus. Diffusion tensor imaging (DTI; van den Heuvel et al., 2008, 2009; Greicius et al., 2009) and resting-state functional Magnetic resonance imaging (fMRI) studies show that these regions are highly interconnected (Greicius et al., 2003; Biswal et al., 2010). The majority of resent studies, that combine resting state fMRI with large-scale “network analyses”, report about functional connectivity, which reflects statistical dependencies (e.g., temporal correlations) between brain regions. But correlation parameter cannot answer the question about causal influence of one neural system on another and we need to find ways towards understanding the effective connectivity. As many investigators report, DMN regions are involved in realization of different tasks, such as introspection and spontaneous cognition (Andrews-Hanna et al., 2010), memory retrieval, emotional process, and social cognition. But the functions of each node within the DMN network are still not clearly understood. And the key idea of how to find out functions of a certain node is to study functionality of those areas to which this node is connected.

There are several methods proposed to measure effective connectivity for fMRI study. They are Structural Equation Modeling (SEM; e.g., Bavelier et al., 2000), the analysis of Granger Causality (GC; e.g., Goebel et al., 2003), and biologically plausible Dynamic Causal Modeling (DCM; see Friston et al., 2003). With the first method it is possible to analyze only steady-state brain connectivity patterns: SEM cannot deal with dynamic changes in fMRI signal. GC is also a model-based approach, where the vector autoregressive model (VAR) is used to assess causal interactions between the fMRI time-series. There are some studies, where GC approach was used to investigate causal relations between the DMN nodes, but their results are hardly consistent. Finally, the third method, DCM, deals with fMRI time series and explicitly models the neuronal dynamics according to underlying effective connectivity. In the course of further development, the opinion starts to prevail that DCM is a more consistent and informative approach to infer causal relationships between brain regions on the basis of fMRI data than others (David et al., 2008; Friston, 2009).

Bayesian approaches are also used to assess connectivity within the DMN. In Wu et al. (2014) it was shown that the DMN could be divided into two subsystems in respect to their functions. These subsystems interact with each other not only in resting-state but also when performing a semantic judgment task. Causal interactions between the key DMN nodes changed dramatically from the resting-state to the task-state. The authors suggest that the DMN could be a stable functional structure both at resting-state and task-state, but under different states the information is processed in the DMN in different ways. The limitation of Bayesian Network approach is that the method cannot discover reciprocal connections of the DMN. It also finds a temporal snapshot of the process and cannot reveal dynamic changes in connectivity between its parts. DCM is free of these limitations and can be supplementary to this study.

DCM is a Bayesian approach typically used to explain effective connectivity changes underlying task-related brain responses (Friston et al., 2003; Sharaev and Mnatsakanian, 2014). When a particular model is specified (including active regions and directed connections between them) connectivity parameters are estimated based on the model structure and the observed fMRI data. Different models are compared using Bayesian Model Selection (BMS; Penny et al., 2004). DCM estimates effective connectivity, which is the measure that mediates the influence that one neuronal system exerts on another. There are many examples of applying DCM to Magnetoencephalography and electroencephalography (MEG/EEG) and fMRI data (see for instance Garrido et al., 2007; Moran et al., 2009; Razi et al., 2015).

DCM treats the brain as a “black box” which receives the input and generates the output. That is why the challenge, when using DCM to study the resting-state network, is that the DCM model cannot be specified without any driving inputs (Stephan and Friston, 2010). For such cases the inclusion of stochastic terms in the model (Daunizeau et al., 2009) might be useful, but one can argue that both spontaneous mental state and fMRI signals during the resting-state are not just random noise. Recently a new version of DCM was introduced based on a deterministic model that generates predicted cross spectra (Friston et al., 2014)—spectral DCM. While stochastic DCM estimates time-dependent fluctuations in neuronal states producing observable fMRI data, spectral DCM evaluates the time-invariant parameters of their cross spectra. This is achieved by replacing the original time series with its second-order statistics (i.e., cross spectra), under stationarity assumptions (Razi et al., 2015). Thus, spectral DCM does not treat neuronal fluctuations as a stochastic noise, and this is why the spectral DCM approach has been chosen for the present study.

The aim of this work is to examine effective connectivity between the main DMN nodes and to estimate the corresponding coupling parameters by applying a novel technique spectral DCM together with modeling resting-state neuronal activity from 30 healthy subjects using Discrete Cosine Set. To test only biologically plausible hypotheses on effective connectivity we used structural connectivity data to reduce the model space.

The resting-state fMRI signals convey fluctuations in the low-frequency band typically within 0.01–0.08 Hz (Biswal et al., 1995), so to identify nodes of the DMN and specify time-series for DCM, the resting state was modeled using a generalized linear model containing a discrete cosine basis set with frequencies ranging from 0.0078–0.1 Hz, in addition to the individual nuisance regressors (Fransson, 2005; Kahan et al., 2014). After this procedure, DCM models were defined, each of them comprising of four main regions of the DMN. The best DCM model was then determined using the BMS procedure as in Stephan et al. (2009).

## Materials and Methods

### Subjects

MRI data was obtained from 30 healthy subjects (10 males and 20 females), mean age 24 (range from 20–35 years). Consent from each participant was provided. The participants were instructed to close their eyes and lie still and relaxed. Each participant was asked about wakefulness during the study; those who fell asleep in scanner would be excluded from the study. Permission to undertake this experiment has been granted by the Ethics Committee of the Institute of Higher Nervous Activity and Neurophysiology of RAS. As for spectral DCM, root mean square error decreases as the number of time points increases; based on results from Razi et al. (2015) we decided to acquire 1000 time points (with a repetition time of 2 s) resulting in approximately 35 min of scanning.

### Scanning Parameters

The MRI data were acquired using a SIEMENS Magnetom Verio three Tesla. The T1-weighted sagittal three-dimensional magnetization-prepared rapid gradient echo sequence was acquired with the following imaging parameters: 176 slices, TR = 1900 ms, TE = 2.19 ms, slice thickness = 1 mm, flip angle = 9°, inversion time = 900 ms, and FOV = 250 × 218 mm^2^. fMRI data were acquired with the following parameters: 30 slices, TR = 2000 ms, TE = 25 ms, slice thickness = 3 mm, flip angle = 90°, and FOV = 192 × 192 mm^2^. Also we received data which contain the options for reducing the spatial distortion of EPI images.

### Imaging Data Analysis

fMRI and anatomical data were pre-processed using SPM12 (available free at http://www.fil.ion.ucl.ac.uk/spm/software/spm12/) based on Matlab. Preprocessing included the following steps: Dicom import, adduction the center of anatomical and functional data to the anterior commissure, reduction of the spatial distortion using Field Map toolbox in spm12 (Friston et al., 2007). Next, slice-timing correction for fMRI data was performed (the correction of hemodynamical response in space and then in time to avoid pronounced motion artifacts; Sladky et al., 2011). Anatomical data were segmented; both anatomical and functional data were normalized. Functional data were smoothed using a Gaussian function with 6 mm isotropic kernel.

We used the SPM toolbox—WFU pickatlas (available free at http://uvasocialneuroscience.com/doku.php?id=uva_socia:wfu_pickatlas) to create a mask for DMN. The mask contains the regions in the right and left hemisphere: Broadman areas 19 and 39, Parahippocampal Gyrus, Posterior Cingulate, Medial Frontal Gyrus (Jann et al., 2010; Di and Biswal, 2014).

The resting state was modeled using a General Linear Model with a discrete cosine basis set (GLM-DCT) consisting of 400 functions with frequencies characteristic to resting state dynamics: 0.0078–0.1 Hz (Biswal et al., 1995; Deco et al., 2011), six nuisance regressors from each session capturing head motion, and the confound time-series from the extra-cerebral compartments. An F-contrast was specified across all frequencies of DCT, producing an SPM that identified regions exhibiting BOLD fluctuations within the frequency band. The obtained statistical parametric maps were then masked by a DMN mask based on previously reported Montreal Neurological Institute (MNI) coordinates for the DMN (Jann et al., 2010; Di and Biswal, 2014). Functional connectivity in DMN is well studied, so for our DCM we took as regions of interest (nodes) most commonly reported four major parts of DMN: the mPFC (3, 54, −2), the PCC (0, −52, 26), left and right intraparietal cortex LIPC (−50, −63, 32) and RIPC (48, −69, 35) (Di and Biswal, 2014). In square brackets there are corresponding MNI coordinates of centers of regions.

**Figure 1:**
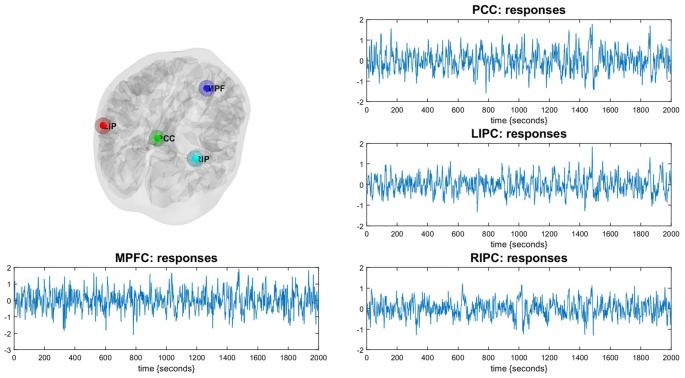
**Illustration of the Default Mode Network (DMN).** The DMN regions are identified using a conventional SPM analysis. Corresponding time-series are principal eigenvariates of regions.

**Figure 2:**
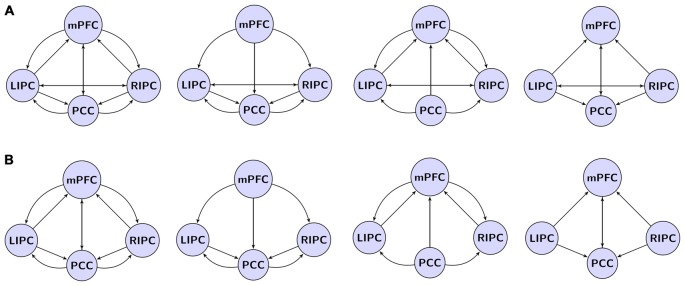
**The investigated model space. (A)** Models with direct connections between bilateral LIPC and RIPC, left to right: full connected model, mPFC, PCC, bilateral modulation. **(B)** Models with no direct connections between LIPC and RIPC. Double arrow means reciprocal connections.

For DCM analysis the principal eigenvariate of a (8 mm radius) sphere was computed (adjusted for confounds) for each region and centered on the peak voxel of the aforementioned F-contrast (see Figure 1). To limit the number of possible models, it was assumed that the model was left right symmetrical. Indeed, no evidence to the contrary was found. The following connectivity models were specified: a full connected model, three models where different regions predominantly affected the other ones (mPFC, PCC and bilateral modulation) and the same models but without direct connections between bilateral LIPC and RIPC, totally 4 × 2 = 8 models, see Figure 2. In order to test model stability in relation to scanning interval, we also constructed the same models (in terms of the DCM nodes and edges) on the first 500 (“initial” model) and last 500 scans (“final” model). After estimating the GLM, one subject did not reveal significant activity in mPFC during the first 500 scans (but did reveal during the last ones), so we had 29 subject for the initial model and 30 for final model.

For each participant these schemes with no exogenous inputs were inverted using spectral DCM. As we assumed that all participants used the same model. Fixed Effects (FFX) BMS (Stephan et al., 2009) was performed to determine the best model which balances the fit of data and the model complexity. Given the best model, the connectivity parameters from each subject were analyzed quantitatively using classical statistics and Bayesian Model Averaging (BMA; Penny et al., 2010), to see whether some of them are stable across a group of subjects. One sample *t*-tests were conducted to examine whether these parameters have significantly nonzero values. In addition, BMA was also conducted to get the probability weighted values of the model parameters. The results of *t*-test and BMA were then compared.

## Results

BMS found the fully connected model to be the best at the group level (eight compared models, Figure 2), consistent with previous similar analyses (Li et al., 2012; Razi et al., 2015). Moreover, this model was the best one for 24 out of 30 subjects.

The fully connected model was the best at the group level for both initial (23 times of 29) and final (26 times of 30) models.

**Figure 3:**
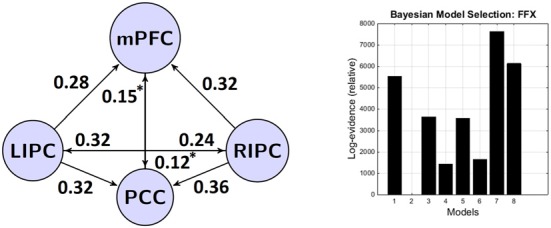
**The winning model at the group level and its connectivity parameters (in Hz).** Left: the winning model and its non-trivial significant (*p* < 0.05) connections. Right: bayesian model selection (BMS) results—models and their (relative) log-evidences. Models legend: 1–lateral modulation with (w) direct connections between bilateral LIPC and RIPC, 2–lateral modulation without (wo) direct connections between bilateral LIPC and RIPC, 3–mPFC modulation (w), 4–mPFC modulation (wo), 5–PCC modulation (w), 6–PCC modulation (wo), 7–full connected (w), 8–full connected (wo). *Non-significant after Bonferroni correction.

The results of BMA and classical *t*-test are shown in the tables below. To concentrate on non-trivial connections, we only report a particular connection if its strength exceeds 0.1 Hz and its probability is greater than 0.95. We also do not consider self-connections in graphs for simplicity. The winning model is shown on Figure 3.

In Tables 1, 2 mean connection strengths are presented (in Hz) from BMA and *t*-test analysis, in Table 3 corresponding standard deviations are shown.

**Table 1:** **Mean connection strengths (in Hz) from BMA**.

BMA	from mPFC	from PCC	from LIPC	from RIPC
to mPFC	0	0.149*	**0.284**	**0.317**
to PCC	0.116*	0	**0.320**	**0.356**
to LIPC	−0.047*	−0.008*	0	**0.321**
to RIPC	−0.044*	−0.045*	**0.249**	0

In rows there are source regions, in columns—target regions. Nontrivial significant (p < 0.05) connections are shown in bold. *Non-significant after Bonferroni correction.

**Table 2:** **Mean connection strengths (in Hz) from *t*-test**.

*t*-test	from mPFC	from PCC	from LIPC	from RIPC
to mPFC	0	0.149*	**0.285**	**0.317**
to PCC	0.116*	0	0.320	**0.356**
to LIPC	−0.046*	−0.009*	0	**0.321**
to RIPC	−0.044*	−0.045*	**0.249**	0

In rows there are source regions, in columns—target regions. Nontrivial significant (p < 0.05) connections are shown in bold. *Non-significant after Bonferroni correction.

**Table 3:** **Standard deviations of connection strengths**.

st. deviation	from mPFC	from PCC	from LIPC	from RIPC
to mPFC	0	0.009	0.013	0.013
to PCC	0.009	0	0.013	0.013
to LIPC	0.007	0.008	0	0.011
to RIPC	0.007	0.008	0.011	0

*In rows there are source regions, in columns—target regions. Standard deviations only for nontrivial significant (p < 0.05) connections are shown*.

From the Tables 1, 2 we can see that BMA and *t*-test results are practically identical, though classical *t*-test does not take into account the estimated precision of each connection, calculated by DCM. So, for further analysis we can use the values obtained from the BMA procedure.

It can be seen that connections from bilateral IPC to mPFC and PCC are rather strong, significant nonzero (*p* < 0.01) and symmetrical in strength (*p* < 0.05). These results are in part similar to those previously reported by Di and Biswal (2014) who found a strong interhemispheric asymmetry in DMN with the emphasis on the right side. Connections between mPFC and PCC are bidirectional, significant in the group and weaker than connections originating from bilateral IPC (*p* < 0.03). In general, all connections from LIPC/RIPC to other DMN regions are much stronger at the group level, so we can assume that these regions have a driving role within the DMN. This result is also similar to the work by Di and Biswal (2014) and Razi et al. (2015).

In Table 4 mean connection strengths are presented (in Hz) for initial/final models respectively, in Table 5 corresponding standard deviations are shown. First, from these tables we can see that the connectivity parameters have, on average, lower values than the parameters of 1000 scans (“total”) model. Especially this could be noticed for LIPC/RIPC connectivity parameters: all of them (except LIPC to PCC connection) are significantly smaller (*p* < 0.05) in initial and final models in comparison to the total model. Between initial and final models these parameters are much alike, though some of them do not pass Bonferroni correction. This could possibly be the result of the shorter scanning time: parameter estimates are less. In Razi et al. (2015) the authors showed root mean square error of parameter estimates to be 50% higher for 512 scans in comparison to 1024. Nevertheless, it can be seen that nontrivial connectivity parameters in total, initial and final models differ only in their magnitude. These differences neither lead to changes in connectivity patterns in terms of existing/absence of a particular connection, nor to changes in roles of a particular connection from being excitatory to inhibitory. This means that the winning model is stable at different time frames in terms of its parameters and reflects relatively stable effective connectivity pattern within the DMN. This pattern may slightly change in time, but the main driving areas and connections among them remain the same. So, we can suggest that the subjects were in approximately the same mental state during first and second half of the experiment.

**Table 4:** **Mean connection strengths (in Hz) for initial (first 500 scans)/final (last 500 scans) models**.

BMA	from mPFC	from PCC	from LIPC	from RIPC
to mPFC	0	0.108*/0.182	0.159/0.131*	0.104*/0.156*
to PCC	**0.166**/0.085*	0	**0.238/0.186**	0.124*/0.205
to LIPC	0.020*/0.024*	0.035*/0.032*	0	0.129*/0.163*
to RIPC	0.036*/0.006*	0.033*/0.040*	0.130*/0.149*	0

In rows there are source regions, in columns—target regions. Nontrivial significant (*p* < 0.05) connections are shown in bold. *Non-significant after Bonferroni correction.

**Table 5:** **Standard deviations of connection strengths for initial (first 500 scans)/final (last 500 scans) models**.

BMA	from mPFC	from PCC	from LIPC	from RIPC
to mPFC	0	0.010/0.010	0.013/0.014	0.014/0.013
to PCC	0.009/0.009	0	0.013/0.013	0.013/0.013
to LIPC	0.008/0.008	0.009/0.009	0	0.011/0.012
to RIPC	0.009/0.008	0.010/0.009	0.012/0.012	0

*In rows there are source regions, in columns—target regions. Standard deviations only for nontrivial significant (p < 0.05) connections are shown*.

It should be noted that despite the fact that the winning model is a fully connected one, not all connection parameters pass the significance threshold. This could be a result of the difference between BMS and BMA. BMS is an inference about models; BMA is an inference about corresponding parameters. In the winning model we may have some parameters that do not individually pass a classical *t*-test, but still contribute to the model in some way. These parameters may have a big variability across subjects, or they can have small values, or both.

## Discussion

We used discrete cosine basis set to model low frequency fluctuations and identify the connection structure underlying the DMN using the spectral DCM on resting-state fMRI data. The best model in a group of 30 subjects suggested strong influence from bilateral IPC to each other and to the mPFC and PCC as well as an information flow from the mPFC to PCC and vice versa.

The authors who studied the coupling between regions within DMN have often reported inconsistent results. For example, Li et al. (2012) using stochastic DCM showed an influence from PCC to mPFC. Other authors (Jiao et al., 2011; Di and Biswal, 2014) using in part different methods such as GCA found a causal influence from mPFC to PCC but not vice versa. We see both connections to be presented in our winning model. Some studies (Jiao et al., 2011; Di and Biswal, 2014) showed that bilateral IPC drives PCC and mPFC, which also is in line with our study. Finally, there is a strong reciprocal connection between bilateral IPC in our best model, also reported by Li et al. (2012). Zhou et al. (2011) found influence from RIPC to LIPC but not vice versa, while authors such as Di and Biswal (2014) and Jiao et al. (2011) did not find any interaction between bilateral IPC. We also found a slight functional asymmetry in bilateral IPC which is an evidence supporting similar findings of Di and Biswal (2014). We found that the bilateral IPC has causal influence on the mPFC and PCC regions, and not vice versa. So we can assume that LIPC and RIPC have a driving or modulating role. This finding is consistent with previous work (Di and Biswal, 2014), partially consistent with Razi et al. (2015) who did not find RIPC effect on the mPFC. However, there are some works with different results, for example, a study using GC (Jiao et al., 2011; Zhou et al., 2011). Zhou et al. (2011) found a causal influence from the LIPC to the mPFC, whereas Jiao et al. (2011) showed symmetrical causal influence from the mPFC to bilateral IPC and from bilateral IPC to PCC.

This discrepancy may be due to a small sample size in previous studies which made some connections not significant or to the different frameworks for measuring causality. In our study we had a rather large sample size (30 subjects) which is enough according to best neuroscience and experimental psychology practices. In addition, one has to note that the DCM framework has been demonstrated empirically to be a more valid method than the GC to study effective connectivity (David et al., 2008).

The mPFC and PCC are two main nodes of the DMN, and are most often reported in different approaches such as Independent component analysis (ICA), seed-based correlations and others. Our results showed a causal influence from the mPFC to PCC, and vice versa. Also, it is worth noting that connections from mPFC to PCC and from PCC to mPFC have practically the same strength and posterior probability.

The PCC has been described as a structural core that links to major brain structures across the whole brain (Hagmann et al., 2008) fulfilling crucial cognitive functions (Kozlovskiy et al., 2012). GC study of whole brain showed that the PCC is a robustly driven hub, which receives information from the whole brain (Deshpande et al., 2011; Yan and He, 2011). Thus, PCC might be a special hub region that collects information from other DMN regions as well as across the whole brain.

The largest proportion of rich club connector hubs was found in the DMN. According to one study, total structural connectivity between peripheral networks with the DMN, salience (Goulden et al., 2014), task-executive networks was dominated by rich club connections in the network. And most connections are linked with peripheral RSN networks.

The high density between DMN, salience, and task-executive networks suggests that PCC can be a meaningful structural and functional core, which accumulates information from cognitive, multimodal networks and may have a very important role in communication with other RSN networks mainly through their representatives in the rich club.

Our results and previous studies show that the PCC is driven by all major regions in the DMN and possibly by other brain regions. From our work and previous studies it remains unclear, however, what are the target regions of information flow out of PCC. One possible hypothesis is that PCC might be a hub between functional networks (Di and Biswal, 2014). This is in line with the theory that the DMN is a higher order cortical system that reciprocally exchanges information with subordinate brain systems (Carhart-Harris and Friston, 2010). Thus, further research is needed to understand how task-specific brain networks depend on DMN.

**Figure 4:**
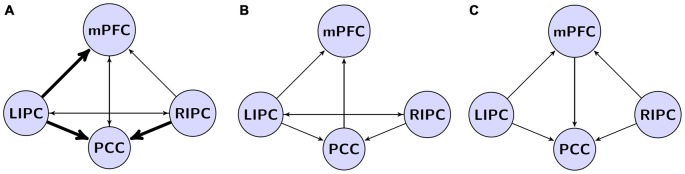
**Our model in a comparison to models from previous studies. (A)** The winning model from current study. Effective connections, common to all existing models are shown by thick arrows. **(B)** The model by Razi et al. (2015) based on spectral dynamic causal modeling (DCM) analysis; **(C)** the model by Di and Biswal (2014) based on deterministic DCM.

Analysis of obtained data shows that the best model has a structure of interactions of DMN regions (marked with thick arrows on Figure 4A) partially similar to the models obtained in other researches (Figures 4B,C), but it also contains connections that integrate other models of interactions (marked with thin arrows on Figure 4A). As in the other models, effective control links of LIPC and RIPC regions are present, connecting each other directly and through mPFC and PCC regions. Obtained data have some differences compared to results of other research groups (see Figure 4), which could be related to change of causal connection weights of neural networks due to active influence of adjunctive regions of DMN. One possible reason for discrepancies between our results and the work by Razi et al. (2015) is the number of scans considered for modeling. When we performed the BMA on initial and final models (first and last 500 scans) some connections (i.e., from RIPC to mPFC) became non-significant after Bonferroni correction. Despite the fact that our connectivity pattern could not be generalized to the whole population with certainty, our findings of pattern stability in time may provide additional evidence of its reliability.

Our next task is to verify stability of the obtained model with the addition of new regions. For example, it is known that PCC region has projection connections with the anterior cingulate, prefrontal, lateral parietal, and para-hippocampal regions (Mantini et al., 2011). It was shown (Mantini and Vanduffel, 2013) that para-hippocampal regions that play a key role in mechanisms of recalling from the memory are also considered as DMN.

We suppose that addition of new functionally important regions such as para-hippocampal cortex can change connections in previously obtained models. This is of particular importance for discussions about the role of DMN networks in modulating cognitive states, as an objective parameter of processes at basic level of neural network functioning (Raichle and Snyder, 2007), the role of mPFC in integration of emotional and cognitive processes (Raichle et al., 2001), and in evaluation of mPFC contributions to mental modeling of behavioral responses based on memory information. Obtained data support hypotheses about involvement of DMN in implementation of two functions—basic spontaneous cognitive level activation (including “top-down” mechanisms) and monitoring of intermodal perception of environmental context (Morcom and Fletcher, 2007; Mantini and Vanduffel, 2013).

It is of particular interest to study causal interactions between large scale networks. For example, whole brain functional connectivity on healthy normal subjects (MEG experiment) has revealed complex network interactions at multiple physiological frequency bands (especially in alpha band; Schmidt et al., 2014). Combination of ICA and DCM was used to confirm the finding of Sridharan et al. (2008) that the Salience Network (SN) is a key for switching between the Central Executive Network (CEN) and the DMN (Goulden et al., 2014). This novel approach to extract the ICA time courses to represent the entire network, rather than to look at regions of interest recently helped us in solving age-old problem of top-down influences in visual perception (Verkhlyutov et al., 2014). In the same vein, it is possible to use DCM examining the relationship between a large numbers of RSNs. In reducing the source space one can come to a better understanding how these networks modulate switching when mental states, task setting or environmental situation are changing.

## Author Contributions

MGS: mathematical methods, programming, data analysis, modeling, article text (introduction, methods, results) and pictures preparation, accountable for all aspects of the work. VVZ: experimental design, data acquisition and preprocessing, final article approval, accountable for all aspects of the work. VLU: work concept, article text (discussion), final article approval, accountable for all aspects of the work. SIK: data acquisition, article text (introduction), final article approval, accountable for all aspects of the work. BMV: overall guidance, project idea, article text (introduction, neuropsychological aspects of discussion), final article approval, accountable for all aspects of the work.

## Funding

This work was partially supported by a grant from the Russian Science Foundation, RScF Project no. 14-28-00234 (structure of DMN and its relation to mental states) and by a grant from the Russian Foundation for Basic Research, RFBR Project ofi-m no. 15-29-01344 (frequency analysis in neurocognitive research).

## Conflict of Interest Statement

The authors declare that the research was conducted in the absence of any commercial or financial relationships that could be construed as a potential conflict of interest.
